# List of reviewers 2025

**DOI:** 10.1192/bjo.2026.10979

**Published:** 2026-02-10

On behalf of the editorial board, the Editor-in-Chief would like to thank the following for their contribution as peer reviewers in the period from January 2025 – December 2025. Their anonymous work for the journal forms the foundation to its success and is highly appreciated.

Karim Abdel Aziz

Mohamed Abdel-Maboud

Oluwakemi Abibu

Elia Abi-Jaoude

Abdullahi Aborode

Achyut Acharya

Daniel Acton

Dimitrios Adamis

Danielle Adams

Anil Adisesh

Gwen Adshead

Demilade Agbeleye

Alastair Ager

Joel Agorinya

Andrea Aguglia

Sayed Ahmadi

Yusuf Akande

Amal Al Fahdi

Nadia Alam

Luis Alameda

Hassen Al-Amin

Steven Albert

Felipe Alckmin

Adam Al-Diwani

Laith Alexander

Regi Alexander

Said Al-Farsi

Farhan Ali

Hafsa Ali

Yumna Ali

John Allan

Judith Allardyce

Janice Allister

Enrique Alonso-Perez

Afsari Banu Alpona

Hasanen Al-Taiar

Hamada Altalib

Mohammed Al-Uzri

Aneline Amalathas

Amanda Amantia Ametaj

Tri Ambarini

Silvia Amoretti

Action Amos

Bhogaraju Anand

Diego Angeles-Valdez

Paul Appelbaum

Rebecca Appleton

Carmelo Aquilina

S.M. Yasir Arafat

Sarah Arias

Urska Arnautovska

Danilo Arnone

Bill Arroyo

Senthil Kumar Arumugam-Subramanian

Ahmad Asgarizadeh

Muqaddas Asif

Muhammad Awais

Jesutofunmi Aworinde

Erland Axelsson

Deji Ayorinde

Agnes Ayton

Ignacio Aznar-Lou

Cate Bailey

Harriet Ball

Jordan Bamford

Marco Bani

Lucy Barker

Rita Barreiros

Barbara Barrett

Tarun Bastiampillai

Joel Bates

Philip Batterham

Joy Noel Baumgartner

Adam Bayes

Ben Beaglehole

Chloe Beale

Peter Beazley

Caragh Behan

Maryam Beiranvand

Valentina Belalcazar Vivas

Sirine Ben Othman

Tomi Bergström

Suze Berkhout

Frank Besag

Ana M. Besoain-Cornejo

Aditya Bharadwaj

Mohan Bhat

Rahul Bhattacharya

Sarmishtha Bhattacharyya

Vishal Bhavsar

Kamaldeep Bhui

Rabia Bibi

Irene Bighelli

Daniel Blackburn

Joseph Boden

Lana Bojanić

Megan Boreham

Rohan Borschmann

Navnita Bose

Emma Boxley

Phillip Boyce

Amy Braddon

Bethany Brand

Christian Brandt

Jaden Brandt

David Branford

Alison Branitsky

E Brocker

Norella Broderick

Elizabeth Brondolo

Guy Brookes

Lisa Brophy

Paola Bucci

Kurt Buhagiar

Claudia Bull

Verónica Raquel Alheia Cabreira

Camilla Cadorin

Ian Callaghan

John Callender

Helen Campbell

Kerem Cantekin

Adelita Cantu

Edoardo Caporusso

Andres Camilo Cardozo Alarcon

Susanna Carella

Anna Carnegie

Mauro Giovanni Carta

Livia Carvalho

Cecilia Casetta

Patricia Casey

Damien Cassells

Giovanni Castellini

Santiago Castiello de Obeso

Andrea Eugenio Cavanna

Yi Chai

Nandini Chakraborty

Shu-Sen Chang

Wing Chung Chang

Iftikhar Ahmed Charan

Samuel Chawner

Hong Chen

Pao-Huan Chen

Wanlin Chen

Koi Man Cheng

Gary Cheung

Carolyn Chew-Graham

Kuo-Liang Chiang

Deborah Chinn

Yujin Choi

Antony Chum

Helgi Clayton Mcclure

Seonaid Cleare

Catherine Clelland

Stephen Clift

Michael Coffey

Erika Comasco

Michael Connors

John Cookson

Marita Cooper

Sally-Ann Cooper

Samuele Cortese

Jose Eduardo Cosentino

Ken Courtenay

Tom Craig

John Crichton

Ioana Cristea

Clair Crockett

Matthew Croucher

Alexis Cullen

Kathryn R. Cullen

Henry Cutler

Rachael Cvejic

Jan Cwik

Nadia Dabbagh

Amber Dadwal

Juliana Dalla

Jayati Das-Munshi

Alice Davis

Jose de Leon

Elena De Rossi

Bertrand De Toffol

Richard Dei-Asamoa

Serhiy Dekhtyar

Valerie Demarinis

Colin Depp

Daniel Derivois

Pushpal Desarkar

Jana DeVilliers

Rashi Dhensa-Kahlon

Sukriti Dhingra

Akshay Dinesh

Roy Dings

Christina Dintica

Sandra Dixon

Bettina K. Doering

Anne M. Doherty

Paula Sinead Donnelly

Diana Dorstyn

Deborah Dover

Alex Dregan

John Drury

Xiangdong Du

Michel Dückers

Anne Duffy

Marie-Michele Dufour

Etienne Duranté

Anna Durbin

Melissa R. Dvorsky

Adisti Dwijayanti

Julian Eaton

Jessica Eccles

Igor Eckert

Clare M. Eddy

Kim Edmunds

Maria Efremova

Carli Ellinghaus

Rebecca Elliott

Timothy Elliott

Deeksha Elwadhi

Esra Emekli

Lidia Engel

Rene Ernst Nielsen

Almila Erol

Whiskey Eromona

Javier Escobar

Fernando Espi Forcen

Amy Essletzbichler

Chris Evans

James Fallon

Lorna Farquharson

Louis Favril

Marc Feger

Emilio Fernandez-Egea

Augusto Ferraris

Mudasir Firdosi

Florian Fischer

Christopher Fitch

Peter Fonagy

Ted Fong

David Foreman

Diego A. Forero

Tomáš Formánek

Michele Fornaro

Andrew Forrester

Sarah Fortune

George Foussias

Joanna Fox

Sophia Frangou

Haojie Fu

Michelle Funk

Frederick Furniss

Rafael Gafoor

Alaa Galadari

Gabriel Galdêncio

Silvana Galderisi

Tim Gale

Yashi Gandhi

Satheesh Kumar Gangadharan

Matt Gaskell

Ruiyang Ge

Christoff Geldenhuys

Raluca Georgescu

Phillip Gerretsen

Frank Gerritse

Dirk Geurts

Raju Ghimire

Baidyanath Ghosh Dastidar

Shreya Godiawala

Shih Ee Goh

Benjamin Goldstein

Fabiano Gomes

Dipesh Gopal

Sasha Gorrell

Kevin Gournay

Karol Grabowski

Nayla Grace

Claire Grant

Lynsey Gregg

Samantha Groves

Marcela Guapacha-Montoya

Matheus Guerra

Rajlakshmi Guha

Mayank Gupta

Nihit Gupta

Siddarth Gupta

Luis Gutierrez-Rojas

Kerry Gutridge

David Guwatudde

Adoración Guzmán García

Peter Haddad

Gareth Hagger-Johnson

Magdalena Hagn

Jessica Hall

Katie Hall

Nutmeg Hallett

Jinming Han

Samuel Hanu

ZHANG Haoran

Hiroyuki Harada

Catherine Harmer

Charlene Harris

Amy Harrison

Yvonne Hartnett

Kenji Hashimoto

Aaron Hauptman

Keith Hawton

Adrian Hayes

Caroline Hayes

Mark Hayward

Adrian Heald

Claire Henderson

Tone Henriksen

Morten Hesse

Sarah Hetrick

Julia Hews-Girard

Thomas Hewson

Fredrik Hieronymus

Nina Higson-Sweeney

Chanler Hilley

Bradley Hillier

Hubertus Himmerich

Anders Hjern

Andreas Hoell

Yaacov Hoffman

Christopher Holmberg

Dawn Holmes

Marcela Horvitz-Lennon

Shabnam Hossein

Tusty-Jiuan Hsieh

Dieneke Hubbeling

Vyv Huddy

Peter Hughes

Vilgot Huhn

Ji-Won Hur

Jonathan Hurlow

Muhammad Husain

İrem Hüzmeli

Aliaa Ibnidris

Tarela Juliet Ike

George Ikkos

Ikbal Inanli

Jeremy Isaacs

Itoro Udo

Ravi Iyer

Henry Jackson

K.S. Jacob

Ricarda Jacob

Brian Jacobs

Irina Jarvers

Syed Javaid

Matthew Jay

Ana Jelovac

Josie Jenkinson

Bumseok Jeong

Zhouxin Jia

Cheryl John

Fabrice Jollant

Brett Jones

Rowena Jones

Michelle Jongenelis

Swati Joshi

Easter Joury

Dan Joyce

Eileen Joyce

Mario Juruena

Duygu Kaba

Ashraf Kagee

Lucie Kalisova

Hanna Kampling

Eila Kankaanpaa

Yesiru Kareem

Azaad Kassam

Cornelius Katona

Caroline Cecil Kaufman

Kenneth Kaufman

F. Kauye

Katerina Kavalidou

Brendan Kelly

Patrick Keown

Kamyar Keramatian

Raihan Khan

Ardavan M. Khoshnood

Reza Kiani

Sarah Kanana Kiburi

Hanna Kienzler

Charles Kim

Daeho Kim

Helena Kim

Minah Kim

Sung-Wan Kim

Taekwan Kim

Gabriella King

David Kingdon

Steve Kisely

Luisa Klahn

Jeroen Knipscheer

Hans Knoblauch

Hans-Helmut Koenig

Douglas Kohler

Rebecca Koncz

Alexander Konnopka

Sandeep Kosaraju

Konstantinos Kotsis

Joshua Kraindler

Vijay Krishnan

Tina Kristensen

Silvia Krumm

Ashish Kumar

Veena Kumari

Satyajit Kundu

Terhi Kurko

Marinos Kyriakopoulos

Kristie Ladergard

Emmeline Lagunes-Cordoba

Yanda Lang

Peter Langdon

Aili Langford

Sylvie Lapierre

Agata Łaszewska

Rachel Latham

James Lathe

Naomi Launders

Stephen Lawrie

Carlo Lazzaro

Lauren Lebois

Ivana Leccisotti

Charles Tzu Chi Lee

Moon-Soo Lee

William Lee

Jeannette Lely

Henna Lemetyinen

Belinda Lennox

Charles Lepkowsky

Chao Li

Jialiang Li

Jiaqi Li

Wenfei Li

Cian-Cian Lin

Hsiang-Yuan Lin

Jialing Lin

Pan Lin

Yu-Kai Lin

Clare Lister

Bangshan Liu

Bao-Peng Liu

Chen-Chung Liu

Feng Liu

Xuebing Liu

Temmy Lo

Maria Loades

Javier-David Lopez-Morinigo

Anikó Lovik

Nasser Loza

Crick Lund

Monty Lyman

Frances Lynch

Stanley Lyndon

Bei Lyu

Sukling Ma

Gabrielle Macaron

Rebecca MacAulay

Orla Macdonald

Lucy Maconick

Julia Magalhães

Venus Mahmoodi

Sloan Mahone

Pallab Majumder

Charles Malpas

Mohammed A Mamun

Kenneth Man

Helen Manley

Zara Mansoor

Jenni Manuel

Belinda Marais

Ruth Marheinecke

Sarah Markham

Peter Martin

Jesús Martín-García

Katie Marwick

Kim Massters

Therese F. Mathisen

Paul Matthews

Chris Maylea

Michelle McCarthy

Andrea Mccloughen

Justin McDaniel

Alexander McFarlane

Joseph F. McGuire

Siobhan McHugh

Colin McKay

Declan McLoughlin

Sally McManus

Sean McNabney

Rupert McShane

Odette Megnin-Viggars

Habtamu Mekonnen

Krakowski Menachem

Ana Paula Mendes-Silva

Paolo Meneguzzo

Adrian Meule

Gisela Mezquida

Wezi Mhango

Christine Miller

Alyssa Milton

Masuma Pervin Mishu

Maria Misiura

Nadav Modlin

Tilaye Moges

Josephine Mollon

Francisco Moreno

Kana Morimoto

Steffen Moritz

Jane Morris

Lee-Anne Morris

Catrin Morrissey

Marina Morrow

Jane Mounty

Di Mu

Armida Mucci

Holger Muehlan

Monika Mueller

Dahlia Mukherjee

Marco Mula

Cornelius Müller

Maurice Mulvenna

Shruti Mutalik

Shinichiro Nakajima

Antor Ndep

Robert A. Neimeyer

Charles B. Nemeroff

Yehuda Neumark

Tamsin Newlove-Delgado

Richard Newton

Qin Xiang Ng

Minh-Hoang Nguyen

Ana Nikcevic

Neil Nixon

Joanne Noblett

Roghieh Nooripour

Alicia Norman

Hilary Norman

Evangelos Ntontis

John-Jose Nunez

Martins Nweke

Jennifer Oates

Aileen O’Brien

Taichi Ochi

Manjula O’Connor

Brian O’Donoghue

David O’Donovan

Omotola Ogunjobi

Kazutaka Ohi

Yasutaka Ojio

Sally O’Keeffe

Dami Okhiria

Emeka Okoli

Miriam Olivola

Claire O’Reilly

Holly O’Rourke

Esteban Ortiz-Prado

Yewande Oshodi

Declan O’Sullivan

Dennis Ougrin

Busra Ozen-Dursun

Sethu Sreekanth

Deborah Padgett

Jon Painter

Milagros Pallavicini

Peter Panayi

Chrysovalantis Papathanasiou

Carmine Pariante

Joel Paris

Jennifer Parker

Stephen Parker

Nasir Pasha

Evgenii Pashnin

Torsten Passie

Stephen Pasternak

Kavitha Pasunuru

Jennifer Payne

Yueheng Peng

Stephanie Penney

Bhathika Perera

Samuel Perry

Eva Petkova

Petros Petrikis

Theodore Petti

Nancy Phillips

Thomas Phillips

Susannah Pick

Bruna Pimentel

Emanuelle Pires

Alexandra Pitman

Oleguer Plana-Ripoll

Vince Polito

Moa Pontén

Rob Poole

Richard Porter

Alex Porthukaran

Stephen Potts

Holly Prigerson

Eleonora Prina

Jesus Privado

Joan Prudic

Kiran Purandare

Andrea Quattrone

Danika Quesnel

Leah Quinlivan

Inti Qurashi

Julia R Pozuelo

Gosia Raczek

Ferose Rahman

Alessandra Raio

Kutlwano Ramaboa

Ravi Ramasamy

Jesus Ramirez-Bermudez

Marya Rana

Natalie Rasgon

Somidha Ray

Gemma Read

John Read

Yansen Alberth Reba

Tobias Redecker

Lennart Reifels

Tai Ren

Sandra Reyes-Beaman

Maryam Riaz

Thomas Richardson

Giulia Rinaldi

Daniel Rivera

Guillermo Rivera

Guillermo Carlos Rivera-Arroyo

Martin Robinson

Lucy Robinson

Deborah Robson

Fernando Rodríguez de Fonseca

Cathryn Rodway

Erin Rogers

Jonathan Rogers

Hans Rohlof

Chayim Rosensweig

Catherine Rothon

Ashok Roy

Marc-André Roy

Meera Roy

Sandor Rozsa

Sonia Ruiz de Azua

David Russ

Marcin Rządeczka

Luigi F Saccaro

Harold Sackeim

Daniel Saddawi-Konefka

Indranil Saha

Laura J. Sahm

Martha Sajatovic

Hawraa Sajwani

Halis Sakiz

Jay Salpekar

Luis Salvador-Carulla

Filipa Sampaio

Conrad Samuelsson

Ignacio Sandia Saldivia

Jacopo Santambrogio

Vedat Sar

Ananya Sarma

Saniye Gökçe Saykal

Steven Schachter

Charlotte Schenk

Marguerite Schneider

Jordan Schriver

Sanne Schuite-Koops

Mohammad A. Seleem

Kathleen Serracino-Inglott

Muhammad Ashir Shafique

Henal Shah

Aparna Shaji

Rohit Shankar

Sifat Sharmin

Carla Sharp

Ben Shaw

Rory Sheehan

Andrew Shepherd

Ashley Brianna Sheppard

Natalie Shoham

Richard Siegert

Justyna Sierpatowska

Ed Silva

Noah Silverberg

Cristiane Silvestre de Paula

Victoria Simms

Gregory Simon

Judit Simon

Alan Simpson

Alexander Simpson

Lindsey Sinclair

Sudhakar Sivapalan

Anna-Kari Sjödin

Theodora Skali

Eric Slade

Karen Slade

Diana Smirnovova

Gregory Smith

John Snowdon

Luke Solomons

Emma Soneson

Natasha Spassiani

Nicolaas Sperna Weiland

Helene Speyer

Anamika Srivastava

Jayesh Srivastava

Helle Stangeland

Jill Stavert

Alberto Stefana

Rosalie Steinberg

Madeleine Stevens

Robert Stewart

Claire Still

Rebecca Strawbridge

Thomas Strouse

Yi Nam Suen

Shuichi Suetani

Kimmo Suokas

Peter Szatmari

Scott Tagliaferri

Ying-Hsuan Tai

Shun Takahashi

Kristiina Tammimies

Celeste Tan

Sandila Tanveer

Timothy Tanzer

Caley Tapp

Liliana Templos Nuñez

Matthew Tennant

Kevin Teoh

Anupam Thakur

Amanda Thomas

Neil Thomas

Charee Thompson

Lindsay Thomson

Nicollette Thornton

Ellen Tingleff

Victoria Tischler

Louise Tofts

Leonardo Tondo

John Torous

Andrea Tortelli

Janet Treasure

Anna Troeger

Samuel Tromans

Dimosthenis Tsapekos

Samson Tse

Apostolos Tsiachristas

Ozlem Kuman Tuncel

Adrià Túnez

Şenol Turan

Massimo Tusconi

Peter Tyrer

Stephen Tyrer

Nian-Sheng Tzeng

Benjamin Underwood

Emanuele Valenti

Zaba Valtuille

Clare van Hamel

Paul Van Houtte

David E. Vance

Cody Varnish

Javiera Vasquez-Nuñez

Krishnaveni Vedavanam

Cindy B. Veldhuis

Pieter Ventevogel

Falk Gerrik Verhees

Gentian Vyshka

Juliet Wakefield

Fredrik Walby

Adrian R. Walker

Sebastian Walther

Haowen Wang

Chaomin Wang

Qiang Wang

Zaid Wani

Sharada Wasti

Philippa Waterhouse

Andrew Watson

Matthew Webb

Shau-Ming Wei

Helen Weiss

Myrna Weissman

Aliza Werner-Seidler

Anna Westermair

Margaret Westwater

Richard Williams

Claire Wilson

Jonathan Wilson

Yun Kwok Wing

Katrina Witt

Miriam Witte

Alan Wong

Roger Wong

Kate Woodcock

Ben Wright

Fay Wright

Eryn Wright

Kai Wu

Jukrit Wungrath

Xijia Xu

Yong Xu

Dharmveer Yadav

Min Yang

Furkan Yazici

Yakup Yildirim

Eren Yıldızhan

Akira Yokoyama

Masafumi Yoshimura

Allan Young

Rwei-Ling Yu

Shuye Yu

Yonggui Yuan

Valerio Zaccaria

Megan Chong Hueh Zan

Hamed Zandian

Nicolò Zarotti

Orestis Zavlis

Francesca Zecchinato

Sana Zehra

Junjie Zhang

Kyann Zhang

Shunming Zhang

Weijun Zhang

Yunshu Zhang

Xiaoyun Zhou

Hannah Ziobrowski

Tessa Zirnsak

Yaara Zisman-Ilani

